# Long term efficacy and toxicity after stereotactic ablative reirradiation in locally relapsed stage III non-small cell lung cancer

**DOI:** 10.1186/s12885-019-5542-3

**Published:** 2019-04-03

**Authors:** Shakeel Sumodhee, Pierre-Yves Bondiau, Michel Poudenx, Charlotte Cohen, Arash O. Naghavi, Bernard Padovani, Daniel Maneval, Jocelyn Gal, Axel Leysalle, Hannah Ghalloussi, Josiane Otto, Jérôme Doyen

**Affiliations:** 1 Department of Radiation Oncology, Centre Antoine-Lacassagne, 33 av de Valombrose, 06189 Nice, France; 2 University of Côte d’Azur, Nice, France; 3 Department of Medical Oncology, Centre Antoine-Lacassagne, 33 av de Valombrose, 06189 Nice, France; 4 Department of Thoracic Surgery, Hôpital Louis-Pasteur, 30 voie romaine, 06000 Nice, France; 5 Department of Radiation Oncology, H. Lee Moffitt Cancer Center and Research Institute, 12902 USF Magnolia, Tampa, Florida, 33612 USA; 6 Department of Radiology, Hôpital Louis-Pasteur 2, 30 voie romaine, 06000 Nice, France; 7 Department of Statistics, Centre Antoine-Lacassagne, 33 av de Valombrose, 06189 Nice, France

**Keywords:** Stage III non-small cell lung cancer, Stereotactic radiotherapy, Reirradiation, Toxicity, Efficacy

## Abstract

**Background:**

In stage III non-small cell lung cancer (NSCLC) treated with concomitant chemoradiotherapy, there is a high rate of relapse. Some of these relapses are only local and can be treated by stereotactic ablative radiation therapy (SABR). Previous studies reporting outcome after SABR reirradiation of the thorax consisted of a heterogeneous population of various lung cancer stages or even different types of cancer. The purpose of study is to evaluate toxicity and outcome of this strategy in locally relapsed stage III NSCLC only.

**Methods:**

From February 2007 to November 2015, 46 Stage III NSCLC patients treated with SABR, for lung recurrence following conventionally fractionated radiation therapy (CFRT), were retrospectively analyzed.

**Results:**

Median follow-up was 47.3 months (1–76.9). The 2 and 4-year progression-free survival (PFS), and overall survival (OS) were of 25.5%/8.6 and 48.9%/30.8%, respectively. Highest presenting toxicity in patients (grade 1 through 5) was: 13 (28.3%), 7 (15.2%), 1 (2.2%), 0 and 2 (4.4%), with deaths due to hemoptysis (*n* = 1) and alveolitis (*n* = 1). Although the Biological Effective Dose (at Planning Tumor Volume isocenter) was lower for central tumors treated for an in-field relapse (*n* = 21, 116 Gy versus 168 Gy, *p* = 0.005), they had no significant difference in OS than the remaining cohort, but with a higher rate of grade 2–5 toxicities (OR = 0.22, [0.06–0.8], *p* = 0.02).

**Conclusion:**

Reirradiation with SABR for local relapse in patients previously treated for stage III NSCLC, is feasible and associated with good outcome. This is also true for central tumors treated for an in-field relapse, but should be radiated with caution to mitigate toxicity.

**Electronic supplementary material:**

The online version of this article (10.1186/s12885-019-5542-3) contains supplementary material, which is available to authorized users.

## Background

Conventionally fractionated radiation therapy (CFRT) with concurrent chemotherapy is the standard of care in patients who have been diagnosed with stage III non-small cell lung cancer (NSCLC) [1], and approximately 40% of these patients will experience intrathoracic recurrences [2]. In this situation, patients may get second-line chemotherapy, salvage surgery, or reirradiation with either CFRT or stereotactic ablative radiotherapy (SABR). The best treatment strategy for intrathoracic recurrences remains unclear, but local treatment is the first choice whenever possible. Previous experience with lung reirradiation with CFRT yields suboptimal 2-years overall survival (OS) of 5–27% with a 5–20% risk of grade ≥ 3 toxicity [3]. SABR is highly effective in the treatment of inoperable stage I lung cancer and has become the standard of care for this group of patients [1]. The effectiveness of SABR arises from the high biologically effective dose (BED) it can achieve in the tumor while maintaining a sharp dose gradient fall off outside the target, preventing dose to critical structures. Higher BED has been associated with improved OS and local tumor control rates in NSCLC [4, 5]. Better dose delivery leads to smaller treatment field and steeper dose drop to organs at risk, which is particularly important in the case of re-irradiation, and can mitigate treatment related sequela. Several studies already reported an acceptable toxicity profile with SABR thoracic reirradiation with 0 to 30% late grade 3–5 pneumonitis, with rare observations of late esophagitis, skin ulceration or fatal hemoptysis [6–15]. In these studies, it is difficult to evaluate outcome because of their short follow-up and patient heterogeneity (previous stage I, II, III or oligometastatic IV disease) [12].

The purpose of the present study is to retrospectively assess toxicity and outcomes of thoracic reirradiation with SABR in patients with locally recurrent NSCLC who were previously treated with CFRT and concurrent chemotherapy limited to stage III NSCLC patients.

## Methods

### Patient selection

With institutional review board approval, we retrospectively reviewed all patients receiving thoracic reirradiation at our institution. We identified all patients with prior history of CFRT for stage III NSCLC, who underwent SABR for local relapse from 02/2007 to 11/2015. All patients were discussed at tumor board and SABR was chosen because patients were not deemed medically operable and because radiofrequency is not considered in previously irradiated patients at our center for safety concerns. Local relapse was determined according to Response Evaluation Criteria in Solid Tumors (RECIST) version 1.1. which is based on imaging findings (CT-scan) [16]. For controversial cases between lung alveolitis and tumor recurrence, we used 18-FDG positron emission tomography-computed tomography and biopsy through lung fibroscopy or CT-scan.

In order to be considered as local recurrence/reirradiation situation, the mass had to be located in the ipsilateral lung and/or in the mediastinum [17] (because previous irradiation was performed with 3 dimensional conformal radiation therapy (3D-CRT) for stage III disease, relapses consequently occurred at least the low/intermediate dose level).

Tumors were considered “central” if they were located < 2 cm from the central airways in any direction (trachea, carina and main bronchus up to the division of the second order bronchi), as per the Radiation Therapy Oncology Group protocol 0813 definition [18].

### Treatment

The treatment protocol for SABR has been previously described in detail [19]. Patients were immobilized with a personalized vacuum cushion. The Cyberknife® (Accuray, Sunnyvale, United States of America) technology was used for the stereotactic treatment. It is characterized by an image-guided, real-time repositioning of the treatment beam during the irradiation session, which is possible after the implantation of gold fiducials into the tumor. Non-coplanar pencil beams using 6MV photons with ray-tracing dosimetric calculation for delivered dose were utilized for all treatments. Dose constraints reported by the AAPM Task Group 101 were used for SABR treatment [20]. There was no dose constraints adaptation regarding the previous received dose, but the following precautions were performed: (i) patients could be treated only if there was a minimal of 6 months delay from their last irradiation; (ii) a GTV / CTV margin and a CTV / PTV margin of 0 mm and 1–2 mm were used, (http://www.cyberknifelatin.com/pdf/brochure-tecnico.pdf), respectively; (iii) the dose was prescribed to the 75–85% isodose line in order to cover the entire PTV volume with 95% of the prescribed dose, and fitted the ICRU-Report 91 for stereotactic radiotherapy; the dose per fraction was decreased if the tumor was centrally located (5 fractions of 10–12 Gy every 2 days instead of 3 consecutive fractions of 20 Gy); (iv) organ at risk protection had to be prioritized over target coverage.

### Follow-up

Patients underwent computed tomographic scan of the thorax and/or positron emission tomography-computed tomography within 7–9 weeks of treatment completion and every 3–6 months thereafter. Tumor response was evaluated according to RECIST 1.1 criteria [16]. Time-to-event outcomes were defined from the last day of SABR. Local control (LC) and locoregional control (LRC) were defined from the last day of SABR to day of local relapse within the irradiated site for LC, and to day of local and/or regional (homolateral thoracic) relapse for LRC. Metastasis-free survival (MFS) was defined as the time to relapse outside the irradiated field. Progression-free survival (PFS) was determined by the time to any relapse (locoregional or metastasis). Overall survival (OS) was defined by the time to death from any cause.

Prior to treatment with SABR, NSCLC patients with a local failure were defined as recurrence after evidence of increased size of enhancing tumor in the treated region. If there were difficulties to assess progression, biopsy through lung fibroscopy was performed. Toxicities were evaluated according to Common Terminology Criteria for Adverse Event (CTCAE) version 4.0. We attempt to contact patients, family or general practitioners in order to collect toxicity and survival data. We could not contact patients who died because of cancer but close medical follow-up was available for them (*n* = 19), and we succeeded in contacting 20 patients (7 (15.2%) patients could not be reached). In this cohort, in-field relapse prior to SABR, was defined as a relapse within the previously irradiated PTV.

### BED calculation

BED was calculated with the following formula: BED = n x d [1 + (d/α/β)], where n = number of fractions, d = dose per fraction and α/β = 10 for non-small lung cancer. BED was evaluated at the Planning Tumor Volume (PTV) isocenter. The minimum BED for the encompassing dose was also reported.

### Statistical analysis

Qualitative data are represented as frequency, percentage and confidence interval 95%. Statistical comparisons were performed using Chi-square tests for categorical data and Mann–U-Whitney’s test for continuous variables. For analysis of quantitative variables, cut-offs were based on the variable’s median value. LC, LRC, MFS, PFS and OS were estimated and presented graphically using the Kaplan Meier method. Patients were censored at the time of death or last follow-up. Survival rates at various times and 95% confidence intervals were also estimated. Log-rank analysis was performed in order to identify potential factors correlated with survival.

All statistical analyses were performed in 5% alpha risk or 95% confidence interval using Statistical Package for the Social Sciences (SPSS) version 16.0 on Windows®. Since premature death may lead to underreporting LC and LRC, competing risk regression model was used to describe LC and LRC survival while considering death as a competing event. Competing risk regression model was also used to determine factors correlated with LC or LRC survival. Subhazard Ratio and 95% confidence intervals were provided for competing risk regression. Stata version 15.1 software was used for these analyses.

## Results

### Characteristics of patients, treatments and lesions (Table 1)

A total of 46 patients were reirradiated with SABR. Twenty-nine (63%) patients were referred by other centers in our country for the treatment of their local relapse. The characteristics of both patients and lesions are shown in Table 1. All patients were treated for a single tumor volume. Tumor board recommended systemic chemotherapy after SABR for 10 patients (Table 1). A second relapse was treated with a second course of SABR for 2 patients while all other relapsed patients were treated with systemic treatment. Seven patients got less than 60 Gy during previous irradiation but were still treated with curative intent because eventually underwent surgery after chemoradiotherapy, due to very good response [21].

**Table 1:** Patient Demographics and Treatment Characteristics

Demographic or Clinical Characteristic	No. of patients	%
Median age, years (range)	66 (44.3–83.3)	
Median follow-up, months (range)	47.3 (1–76.9)	
Gender		
Male	35	76.1
Female	11	23.9
Histology		
Squamous	22	47.8
Non-squamous	24	52.2
Performans Status - ECOG^a^		
0	27	71.1
1	11	28.9
Missing	8	
Location of relapse		
Central	24	52.2
Peripheral	22	47.8
In-field relapse		
Yes	29	63
No	17	37
Primary stage (at first irradiation)		
IIIA	21	45.7
IIIB	25	54.3
Concurrent chemotherapy at first irradiation		
Yes	35	76.1
No	11	23.9
Surgery immediately after the first irradiation		
Yes	11	23.9
No	35	76.1
First irradiation		
Median total dose	66 Gy (44–70)	
Median dose per fraction	2 Gy (1.8–2.3)	
Total dose < 60 Gy	7 patients	
Median BED dose	79.2 Gy (39–90)	
Concomitant chemotherapy (first irradiation)		
Cisplatin - Docetaxel	17	54.8
Carboplatin – Paclitaxel	3	9.7
Cisplatin – Navelbine	3	9.7
Carboplatin – Etoposide	2	6.5
Carboplatin - Docetaxel	2	6.5
Cisplatin – Paclitaxel	1	3.2
Cisplatin – Etoposide	1	3.2
Carboplatin – Gemcitabine	1	3.2
Carboplatin - Navelbine	1	3.2
Unknown	4	
Reirradiation		
Median delay between irradiations	22.6 (6.2–101.5)	
Median tumor size in mm	33 (10–60)	
Median GTV in mL	13.2 mL (1.1–79.1)	
Median duration of treatment in days	5 (3–12)	
Median total physical prescribed dose in Gy	60 (40–75)	
Median prescribed dose per fraction in Gy	16 (10–20)	
Median number of sessions	4 (3–5)	
Median BEDisocenter in Gy	132 (72–187.5)	
Median BED in Gy	50.2 (6.7–189.1)	
Isodose of prescription in %	80 (70–83)	
Median GTV coverage in %	95.6 (43.8–100)	
Median PTV coverage in %	90.2 (34.8–100)	
Adjuvant chemotherapy after reirradiation		
Yes^a^	10	21.7
No	36	78.3

^a^Adjuvant chemotherapy consisted in platinum based chemotherapy in 6 and pemetrexed alone in 4 patients

Median size of reirradiated tumors was 33 mm (10–60). The median time from primary CFRT treatment to reirradiation with SABR was 22.6 months (6.2–101.5). Type of concomitant chemotherapy is reported in Table 1.

### Response and survival data (Table 2, Figs. 1 and 2)

There were 10 (21.7%) complete responses (CR), 19 (41.3%) partial responses (PR), 14 (30.4%) stabilized disease (SD) and 3 (6.5%) immediate local progressive disease (PD). Improvement in the CR and PR rate was associated with tumor size < 33 mm, patients treated for an out-of-field relapse, and PTV coverage ≥90%. On multivariate analysis, only tumor size independently predicted for tumor response (Table 2).

**Table 2:** Predictive factors for tumor response

Variable	Complete or Partial Response	Stabilization or progression	*p* value (Chi Square)	Binary logistic regression^b^	*p*-value
Gender					
Male (*n* = 35)	60%	40%	0.4	NI	
Female (*n* = 11)	72.7%	27.3%			
Histology					
Squamous (*n* = 22)	59.1%	40.9%	0.6	NI	
Non squamous (*n* = 24)	66.7%	33.3%			
Age					
< 66 years (*n* = 23)	69.6%	30.4%	0.3	NI	
≥ 66 years (*n* = 23)	56.5%	43.5%			
Dose of reirradiation					
BED ≤130 Gy (*n* = 18)	50%	50%	0.14	NI	
BED > 130 Gy (*n* = 28)	71.4%	28.6%			
BEDmin ≤50 Gy (*n* = 22)	54.5%	45.5%	0.3	NI	
BEDmin > 50 Gy (*n* = 23)	69.6%	30.4%			
Tumor size					
≥ 33 mm (*n* = 21)	42.9%	57.1%	0.008	1	0.04
< 33 mm (*n* = 22)	81.8%	18.2%		OR = 0.21	
Missing value (*n* = 3)				[0.04–0.93]	
GTV Volume					
≥ 13 mL (*n* = 22)	33.3%	76.5%	0.005	NI	
< 13 mL (*n* = 22)	66.7%	23.5%			
Missing value (*n* = 2)					
SABR duration					
≥ 6 days (*n* = 25)	64%	36%	0.8	NI	
< 6 days (*n* = 21)	61.9%	38.1%			
Number of fractions					
> 3 (*n* = 30)	60%	40%	0.5	NI	
≤ 3 (*n* = 16)	68.8%	31.2%			
Dose per fraction					
> 12 (*n* = 23)	69.6%	30.4%	0.3	NI	
≤ 12 (*n* = 16)	56.5%	43.5%			
Performans Status ECOG^a^					
0 (*n* = 27)	70.4%	29.6%	0.9	NI	
1 (*n* = 11)	72.7%	27.3%			
Missing value (*n* = 8)					
Location of relapse					
Central (*n* = 24)	50%	50%	0.06	NI	
Peripheral (*n* = 22)	77.3%	22.7%			
Primary stage (at first irradiation)					
IIIA (*n* = 21)	61.9%	38.1%	0.9	NI	
IIIB (*n* = 25)	64%	36%			
In-field relapse					
Yes (*n* = 29)	48.3%	51.7%	0.03	1	0.14
No (*n* = 17)	82.4%	17.6%		OR = 0.26 [0.04–1.6]	
In-field relapse and central tumors					
Yes (*n* = 19)	35.3%	64.7%	0.01	NI^c^	
No (*n* = 26)	72.4%	27.6%			
GTV coverage					
< 95% (*n* = 22)	50%	50%	0.07	NI	
≥ 95% (*n* = 24)	75%	25%			
PTV coverage					
< 90% (*n* = 23)	47.8%	52.2%	*p* = 0.03	1	0.11
≥ 90% (*n* = 23)	78.3%	21.7%		OR = 3.2 [0.74–13.8]	

^a^At time of stereotactic ablative radiotherapy; ECOG = Eastern Cooperative Oncology Group

^b^Complete-Partial response versus stabilization-progression

^c^If “in-field relapse and central tumors” is included in multivariate model instead of “in-field relapse”: *p*-value for “in-field relapse and central tumors” = 0.08 (0.03 for tumor size and 0.15 for PTV coverage)

**Fig. 1:**
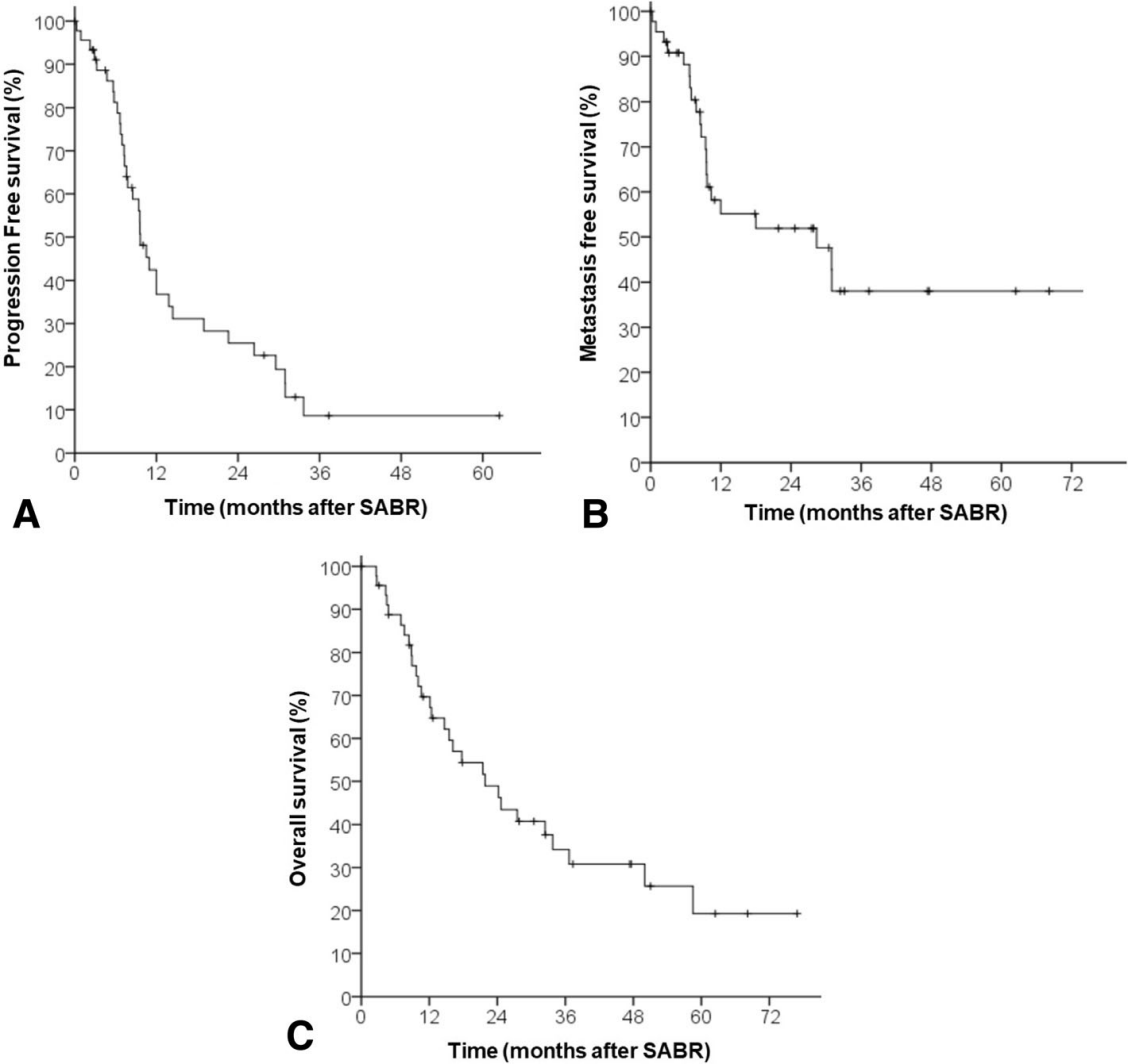
Progression-Free Survival (**a**), Metastasis-Free Survival (**b**), and Overall Survival (**c**)

**Fig. 2:**
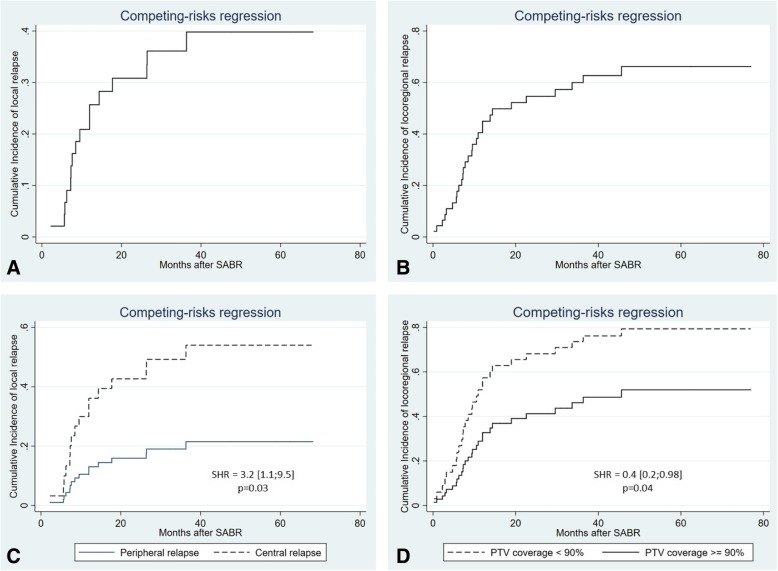
Competing risk regression of local relapse (**a**), locoregional relapse (**b**), local relapse as a function of relapse location (**c**), locoregional relapse as a function of tumor coverage (**d**)

With a median follow up of 47.3 months (1–76.9), 34 patients experienced a relapse (16 local relapse, 13 regional relapse, 21 metastatic relapse), and 29 patients died. The Kaplan-Meier estimated 4-year LC and LRC rate were 44.2 and 14.7%, respectively. Competing risk analysis identified a 4-year rate of local relapse and locoregional relapse of respectively 40 and 67% (Fig. 2). The 4-year PFS, MFS and OS rate were 10.8, 30.8 and 30.8%, respectively (Fig. 1). The median LC, LRC, MFS, PFS and OS were 36.3 months (2.2–68.2), 13.8 months (1;76.9), 28.3 months (1–76.9), 9.6 (1–62.5) and 21.8 months (2.6–76.9), respectively (Kaplan Meier). Of note, the cause of death was either not related to cancer or unknown in 9 of 29 patients. Among these 9 patients, there were two treatment related death, 3 lethal documented lung infection, and 4 deaths from unknown cause. For these 4 patients there was no relapse at last follow-up.

### Predictive factors for LC, LRC, MFS, PFS and OS (Tables 3 and 4)

On univariate analysis, the location of relapse was associated with worse local control, specifically disease in the central thorax (SHR: 3.2 [1.1;9.5], Fig. 2c).

**Table 3:** Prognostic factors for local control (LC), Locoregional control (LRC) and metastasis-free survival (MFS)

	Local relapse		Locoregional relapse	
Variable	Subhazard Ratio (SHR, 95% CI)	*p*-value	Subhazard Ratio (SHR, 95% CI)	*p*-value
Gender				
Male (*n* = 35)	1	*p* = 0.2	1	*p* = 0.5
Female (*n* = 11)	0.5 [0.1–1.6]		0.7 [0.3–1.9]	
Histology				
Non squamous (*n* = 24)	1	*p* = 0.3	1	*p* = 0.9
Squamous (*n* = 22)	1.48 [0.6–3.2]		0.9 [0.5–1.7]	
Age				
< 66 years (*n* = 23)	1	*p* = 0.4	1	*p* = 0.9
≥ 66 years (*n* = 23)	1.4 [0.5–3.7]		0.9 [0.5–2]	
Period between irradiations				
> 24 months (*n* = 20)	1	*p* = 0.8	1	*p* = 0.1
≤ 24 months (*n* = 25)	1.1 [0.4–2.9]		1.8 [0.8–3.9]	
Missing value (*n* = 1)				
> 12 months (*n* = 36)	1	*p* = 0.8	1	*p* = 0.5
≤ 12 months (*n* = 9)	1.09 [0.3–3.6]		1.3 [0.5–3.5]	
Missing value (*n* = 1)				
Dose of reirradiation				
BED > 130 Gy (*n* = 28)	1	*p* = 0.9	1	*p* = 0.09
BED ≤130 Gy (*n* = 18)	0.9 [0.3–2.5]		1.8 [0.9–3.9]	
BEDmin ≤50 Gy (*n* = 22)	1	*p* = 0.16	1	*p* = 0.06
BEDmin > 50 Gy (*n* = 23)	0.5 [0.2–1.3]		0.5 [0.2–1.05]	
SABR duration				
≥ 6 days (*n* = 25)	1	*p* = 0.4	1	*p* = 0.7
< 6 days (*n* = 20)	1.5 [0.56–4.11]		0.9 [0.4–1.8]	
Chemotherapy after reirradiation				
Yes (*n* = 10)	1		1	
No (*n* = 36)	1.6 [0.4–5.5]	*p* = 0.4	1.6 [0.7–3.9]	*p* = 0.2
Tumor size				
< 33 mm (*n* = 22)	1		1	
≥ 33 mm (*n* = 21)	1.58 [0.6–4.1]	*p* = 0.3	1.6 [0.8–3.5]	*p* = 0.18
Missing value (*n* = 3)				
GTV Volume				
< 13 mL (*n* = 22)	1		1	
≥ 13 mL (*n* = 22)	1.49 [0.57–3.95]	*p* = 0.4	1.83 [0.87–3.82]	*p* = 0.10
Missing value (*n* = 2)				
SABR duration				
≥ 6 days (*n* = 25)	1	*p* = 0.4	1	*p* = 0.7
< 6 days (*n* = 21)	1.51 [0.56–4.1]		0.87 [0.42–1.79]	
Number of fractions				
≤ 3 (*n* = 16)	1	*p* = 0.09	1	*p* = 0.9
> 3 (*n* = 30)	2.85 [0.85–9.6]		0.99 [0.47–2.1]	
Dose per fraction				
> 12 (*n* = 23)	1	*p* = 0.8	1	*p* = 0.2
≤ 12 (*n* = 22)	0.93 [0.35–2.44]		1.54 [0.74–3.21]	
Performans Status - ECOG^a^				
1 (*n* = 11)	1	*p* = 0.7	1	*p* = 0.19
0 (*n* = 27)	0.78 [0.2–2.9]		1.8 [0.7–4.4]	
Missing value (*n* = 8)				
Location of relapse				
Peripheral (*n* = 22) Central	1	*p* = 0.03	1	*p* = 0.9
(*n* = 24)	3.2 [1.1–9.5]		0.9 [0.4–2.1]	
Primary stage (at first irradiation)				
IIIA (*n* = 21)	1	*p* = 0.6	1	*p* = 0.11
IIIB (*n* = 25)	1.2 [0.5–3.2]		1.8 [0.9–3.7]	
In-field relapse				
No (*n* = 17)	1	*p* = 0.2	1	*p* = 0.2
Yes (*n* = 29)	2 [0.7–5.9]		1.6 [0.7–3.6]	
In-field relapse and central tumors				
No (*n* = 26)	1	*p* = 0.04	1	*p* = 0.8
Yes (*n* = 19)	2.8 [1.1–7.3]		0.9 [0.4–1.9]	
Adjuvant chemotherapy				
No (*n* = 36)	1	*p* = 0.4	1	*p* = 0.2
Yes (*n* = 10)	1.6 [0.5–5.5]		1.6 [0.7–3.8]	
GTV coverage				
< 95% (*n* = 22)	1	*p* = 0.1	1	*p* = 0.07
≥ 95% (*n* = 24)	0.45 [0.17–1.18]		0.5 [0.2–1.06]	
PTV coverage				
< 90% (*n* = 23)	1	*p* = 0.17	1	*p* = 0.04
≥ 90% (*n* = 23)	0.5 [0.19–1.32]		0.4 [0.2–0.98]	

^a^At time of stereotactic ablative radiotherapy; ECOG = Eastern Cooperative Oncology Group

**Table 4:** Prognostic factors for progression-free survival (PFS) and overall survival (OS)

Variable	2-yearMFS	Log-Rank	2-year PFS	Log-Rank	2-year OS	Log-Rank
Gender						
Male (*n* = 35)	38.6%	*p* = 0.4	29.5%	*p* = 0.02	49.2%	*p* = 0.9
Female (*n* = 11)	55.1%		11.3%		45.7%	
Histology						
Squamous (*n* = 22)	41.3%	*p* = 0.5	28.6%	*p* = 0.7	31.1%	*p* = 0.2
Non squamous (*n* = 24)	60.9%		22.5%		66.5%	
Age						
≥ 66 years (*n* = 23)	59.2%	*p* = 0.7	22.5%	*p* = 0.3	31.3%	*p* = 0.02
< 66 years (*n* = 23)	47.6%		27.3%		65.7%	
Period between irradiations						
> 24 months (*n* = 20)	50.9%	p = 0.6	18.2%	p = 0.5	69.3%	p = 0.11
≤ 24 months (*n* = 25)	55.6%		33.4%		37.9%	
Missing value (*n* = 1)						
> 12 months (*n* = 36)	59.7%	*p* = 0.3	27.9%	*p* = 0.3	59.5%	*p* = 0.07
≤ 12 months (*n* = 9)	21.4%		18.8%		12.7%	
Missing value (*n* = 1)						
Dose of reirradiation						
BED ≤130 Gy (*n* = 18)	61.1%	*p* = 0.15	28.3%	*p* = 0.2	47.9%	*p* = 0.8
BED > 130 Gy (*n* = 28)	44.4%		23%		49.6%	
BEDmin ≤50 Gy (*n* = 21)	53%	*p* = 0.3	21.2%	*p* = 0.7	52.9%	*p* = 0.4
BEDmin > 50 Gy (*n* = 23)	46.6%		32.2%		42.2%	
SABR duration						
≥ 6 days (*n* = 25)	51.9%	*p* = 0.3	21.6%	*p* = 0.3	53%	*p* = 0.6
< 6 days (*n* = 20)	54%		30.3%		45.2%	
Number of fractions						
> 3 (*n* = 30)	50.7%	*p* = 0.5	17.5%	*p* = 0.4	47.1%	*p* = 0.6
≤ 3 (*n* = 16)	56.6%		40.8%		52.1%	
Dose per fraction						
> 12 (*n* = 23)	45.7%	*p* = 0.4	26.2%	*p* = 0.6	49%	*p* = 0.6
≤ 12 (*n* = 22)	58.8%		24.2%		49.1%	
Chemotherapy after reirradiation						
Yes (*n* = 10)	50.8%	*p* = 0.9	19%	*p* = 0.5	38.9%	*p* = 0.4
No (*n* = 36)	52.6%		27%		51.3%	
Tumor size						
≥ 33 mm (*n* = 21)	42.5%	*p* = 0.2	13.7%	*p* = 0.2	31.7%	*p* = 0.059
< 33 mm (*n* = 22)	58.9%		31%		69.8%	
Missing value (*n* = 3)						
GTV Volume						
≥ 13 mL (*n* = 22)	38.4%	*p* = 0.12	13.1%	*p* = 0.10	27.3%	*p* = 0.06
< 13 mL (*n* = 22)	58.9%		31%		70.6%	
Missing value (*n* = 2)						
Performans Status - ECOG^a^						
0 (*n* = 27)	57.4%	*p* = 0.3	34.2%	*p* = 0.6	59.9%	*p* = 0.4
1 (*n* = 11)	60.6%		21.8%		66.3%	
Missing value (*n* = 8)						
Location of relapse						
Central (*n* = 24)	46.7%	*p* = 0.9	16.5%	*p* = 0.4	43.1%	*p* = 0.2
Peripheral (*n* = 22)	59%		35.1%		57.5%	
Primary stage (at first irradiation)						
IIIA (*n* = 21)	59.7%	*p* = 0.3	30.6%	*p* = 0.3	49.2%	*p* = 0.3
IIIB (*n* = 25)	44.4%		21.2%		48.6%	
In-field relapse						
Yes (*n* = 29)	47.4%	*p* = 0.4	18.3%	*p* = 0.11	42.4%	*p* = 0.11
No (*n* = 17)	58.8%		37%		61.1%	
In-field and central tumors						
Yes (*n* = 19)	45.3%	*p* = 0.9	14.2%	*p* = 4	36.1%	*p* = 0.09
No (*n* = 27)	57%		33%		60.8%	
Adjuvant chemotherapy						
No (*n* = 36)	52.6%	*p* = 0.8	27%	*p* = 0.5	51.3%	*p* = 0.4
Yes (*n* = 10)	50.8%		19%		38.9%	
GTV coverage						
< 95% (*n* = 22)	48.9%	*p* = 0.8	19%	*p* = 0.6	47.2%	*p* = 0.7
≥ 95% (*n* = 24)	54.4%		33.3%		49.9%	
PTV coverage						
< 90% (*n* = 23)	46.6%	p = 0.9	18.2%	p = 0.5	44.9%	*p* = 0.9
≥ 90% (*n* = 23)	58.1%		35.6%		52.4%	

^a^At time of stereotactic ablative radiotherapy; ECOG = Eastern Cooperative Oncology Group

Of note, the BED (at PTV isocenter) delivered to central tumors was lower than the BED delivered to peripheral tumors (116 Gy versus 180 Gy, *p* = 0.004), as well was the BED delivered to central tumors with an in-field relapse in comparison with others (113 Gy vs 150 Gy, *p* = 0.007). Tumor size, Gross Tumor Volume (GTV) and PTV coverage did not differ between peripheral and central tumors (*p* = 0.06, *p* = 0.11, *p* = 0.4, respectively).

PTV coverage was the unique predictive factor for locoregional relapse rate (lower rate of locoregional relapse if coverage ≥90%: SHR = 0.4 [0.2;0.98], *p* = 0.04, Fig. 2d).

No factors correlated with MFS. Male presented with improved PFS in univariate analysis (2-year PFS: 29.5% versus 11.3%, *p* = 0.02).

The only factor associated with OS was age (age < 66 years was correlated with improved 2-year OS rate: 65.7% vs 31.3%, p = 0.02). There was a trending 2-year OS detriment associated with larger tumor size ≥33 mm (31.7% vs 69.8%, *p* = 0.059), interval to reirradiation < 12 months (12.7% vs 59.5%, *p* = 0.07) and in-field relapse (40% vs 74.5%, *p* = 0.06).

### Toxicity profile (Table 5)

Side effects after SABR were reported by 24 (52.2%) patients. Respiratory symptoms were observed in 12 patients (26.1%) and one patient died from radiation alveolitis. Thirteen (28.3%), 7 (15.2%), 1 (2.2%), 0 and 2 (4.4%) patients presented with a maximum toxicity grade of 1, 2, 3, 4 and 5, respectively. One patient died because of hemoptysis and another, as already mentioned, from radiation alveolitis. Additional file 1: Table S1 describes characteristics of the 2 patients who experienced fatal toxicities. Of note, the patient with grade 5 hemoptysis presented with an elevated dose to the proximal bronchial vascular tree.

**Table 5:** Toxicities

Toxicity	Grade 1	Grade 2	Grade 3	Grade 4	Grade 5
Asthenia	3	3	0	0	0
Alveolitis	5	1	0	0	1
Pneumonitis	0	1	0	0	0
Dysphonia	0	1	0	0	0
Cough	2	0	0	0	0
Hemoptysis	0	0	0	0	1
Esophagitis	1	1	0	0	0
Pain	2	0	0	0	0
Rib fracture	0	1	0	0	0
Breast cancer	0	0	1	0	0

The 2 patients treated with 2 SABR reirradiations had no grade 3 or more toxicities. One patient presented with second primary breast cancer requiring hospitalization; this may be a radiation associated malignancy and was consequently classified as a grade 3 toxicity.

### Predictive factors for grade 2–5 toxicities (Table 6)

On univariate analysis, predictive factors for a higher rate of grade 2–5 toxicities were tumor size ≥33 mm, central tumors and in-field relapse. Central tumors and in-field relapse had a higher rate of grade 2–5 toxicities. Initial multivariate analysis showed no factors associated with toxicity. Replacing the location of relapse (central vs. peripheral) and whether the tumor was an in-field relapse at the time of SABR with a variable incorporating both risks (in-field relapse and central vs. other), was independently predictive of grade 2–5 toxicity on multivariate analysis.

**Table 6:** Predictive factors for grade 2 to 5 toxicities

Variable	Rate of G2–5 toxicities	Chi Square	Binary logistic regression	*p*-value	Binary logistic regression	*p*-value
Gender						
Male (*n* = 35)	25.7%	*p* = 0.2	NI		NI	
Female (*n* = 11)	9.1%					
Age						
≥ 66 years (*n*= 23)	30.4%	*p* = 0.15	NI		NI	
< 66 years (*n* = 23)	13%					
Period between irradiations						
> 24 months (*n* = 20)	20%	*p* = 0.7	NI		NI	
≤ 24 months (*n* = 25)	24%					
Missing value (*n* = 1)						
> 12 months (*n* = 36)	16.7%	*p* = 0.07	NI		NI	
≤ 12 months (*n* = 9)	44.4%					
Missing value (*n* = 1)						
Dose of first irradiation						
BED < 76 Gy (*n* = 18)	22.2%	*p* = 0.9	NI		NI	
BED ≥76 Gy (*n* = 28)	21.4%					
Dose of reirradiation						
BED ≤130 Gy (*n* = 18)	27.8%	*p* = 0.4	NI		NI	
BED > 130 Gy (*n* = 28)	17.9%					
Tumor size						
≥ 33 mm (*n* = 21)	38.1%	*p* = 0.02	1	*p* = 0.10	1	*p* = 0.09
< 33 mm (*n* = 22)	9.1%		OR = 0.22		OR = 0.21	
Missing value (*n* = 3)			[0.04–1.36]		[0.04–1.2]	
GTV Volume						
≥ 13 mL (*n* = 22)	36.4%	*p* = 0.03			NI	
< 13 mL (*n* = 22)	9.1%		NI			
Missing value (*n* = 2)						
SABR duration						
≥ 6 days (*n* = 25)	36%	*p* = 0.8	NI		NI	
< 6 days (*n* = 21)	38.1%					
Number of fractions						
> 3 (*n* = 30)	26.7%	*p* = 0.2	NI		NI	
≤ 3 (*n* = 16)	12.5%					
Dose per fraction						
> 12 (*n* = 23)	13%	*p* = 0.15	NI		NI	
≤ 12 (*n* = 16)	30.4%					
Performans Status - ECOG *						
0 (*n* = 27)	22.2%	*p* = 0.3	NI		NI	
1 (*n* = 11)	9.1%					
Missing value (*n* = 8)						
Location of relapse						
Central (*n* = 24)	37.5%	*p* = 0.007	1	*p* = 0.07	1	*p* = 0.03
Peripheral (*n* = 22)	4.5%		OR = 0.12 [0.01–1.17]		OR = 6.4 [1.1–37.7]	
In-field relapse and central tumors						
No (*n* = 27)	7.4%	*p* = 0.005	NI		NI	
Yes (*n* = 19)	42.1%					
Primary stage (at first irradiation)						
IIIA (*n* = 21)	28.6%	*p* = 0.3	NI		NI	
IIIB (*n* = 25)	16%					
In-field relapse						
Yes (*n* = 29)	31%		1	*p* = 0.3		
No (*n* = 17)	5.9%	*p* = 0.04	OR = 0.33 [0.03–3.49]			

## Discussion

This study reports the outcome of patients previously treated with CFRT for stage III NSCLC, and reirradiated with SABR because of lung recurrence.

At last follow-up, 32 patients had relapsed (16 local, 13 regional and 21 metastatic relapse), and 29 patients died, leading to a 2 and 4-year PFS and OS of 26.1%/10.8 and 48.9%/30.8%, respectively (Kaplan-Meier). Thus, approximately one-third of patients were alive 4 years after SABR but only 10.8% free of relapse, emphasizing that in addition to local control, distant control is concerning for this population. Although adjuvant chemotherapy was not prognostic in the present study (Tables 3 and 4), it was likely underpowered to address this question. The latest National Comprehensive Cancer Network guidelines (NCCN version 3.2019) regarding local recurrence in NSCLC advises reresection or SABR with no clear recommendation on the treatment thereafter (observation or systemic therapy). To our knowledge, there is no study which analysed the impact of adjuvant chemotherapy after the treatment of local recurrence in stage III NSCLC. However, regarding the high rate of relapse following SABR reirradiation, adjuvant systemic treatment (chemotherapy, immunotherapy) may be recommended. Newer strategies could combine SABR and immunotherapy, as the SABR may improve immunotherapy efficacy, as described in the recent randomized phase II trial for advanced NSCLC [22].

In our study, age was the only variable associated with OS. Otherwise, there were no factors significantly correlated with MFS, PFS or OS, and interestingly central tumors treated with SABR for an in-field relapse had no significant difference in OS, when compared to the remaining cohort. In this subgroup there was also a significant increase of grade 2–5 toxicities (42.9% versus 4.5%, *p* = 0.007). This consequently suggests that patients with central and in-field relapse are also good candidate for SABR reirradiation. Of note central tumors (*n* = 24) presented with a higher risk of local relapse suggesting that the absence of LC difference between central tumors treated for an in-field relapse (*n* = 21) with others, may be due to a lack of statistical power.

In the present cohort the cause of death was not only cancer related but also to treatment related toxicities (*n* = 2), lung infection (*n* = 3) and unknown cause (*n* = 4). A recent study found that cause of death in locally advanced NSCLC was mainly cancer related, but 3 years after diagnosis, the proportion of death related to cardiovascular disease, other cancer, chronic obstructive pulmonary disease, increases up to 25–40% [23]. Managing non-cancer related morbidity is still important in this frail population.

Previous studies analyzing SABR reirradiation on the thorax did find similar results as our study, with good survival and acceptable toxicity profile, but included a heterogeneous patient population, including patients of all stages, with locoregional or metastatic relapse, and patients previously treated by CFRT or SABR.

By limiting our scope to stage III NSCLC, our study is one of the first to analyze the outcome of SABR reirradiation in patients with stage III at first diagnosis, with the largest follow-up (47 months) and largest population (*n* = 46) to date. A smaller series that is similar to our current study, reported 17 patients reirradiated for in-field relapse and noted a 1-year OS of 59% and a 1-year LC of 86%, but with a 12% fatal toxicity rate (median follow-up = 18 months) [10]. Kelly et al described the treatment of 37 patients (initial NSCLC stage: I to IV) with a median follow-up of 15 months: there were 19% grade 3 alveolitis and 8% grade 3 esophagitis [24]. Liu et al reported pulmonary toxicities of 72 patients treated with reirradiation (median follow-up = 16 months), however the prior thoracic irradiation included NSCLC, small-cell lung cancer and esophageal cancer [14]. Other reirradiation series describe a 1-year LC rate ranging between 65 and 77%, none of which analyzed a homogeneous population of stage III NSCLC patients [9, 12, 15, 25]. Prognostic factors for better outcome in the context of locally relapse stage III NSCLC are unknown, In patients with re-irradiated for NSCLC, Kelly et al found that out-of-field recurrences had a better PFS [24], but the patient’s initial NSCLC stage ranged from I-IV. In the present study, good candidates for SABR reirradiation in locally relapsed stage III NSCLC patients includes: peripheral tumors, tumor size < 33 mm (or 13 mL), and an interval between irradiation and in-field relapse ≥12 months. Larger cohorts are required to further delineate the prognostic factors in this population. Toxicity after thoracic reirradiation continues to be a concern. A pooled analysis of 14 studies evaluating toxicity after high-dose reirradiation of NSCLC found mean rates of 7% for pulmonary toxicity grades ≥3. Grade 5 lethal bleeding was observed in 12 of 408 patients (3%) [7]. Therefore, it is important to mitigate these toxicities by reducing prescription dose, tumor coverage (to protect proximal bronchial vascular tree (PBV) and lung) or increasing the number fractions. Dose constraints in this setting are difficult to establish when combining a conventional and hypofractionated radiotherapy regimen. Hepel et al identified that 1 cc and 4 cc of PBV should not receive more than a mean dose of 20 Gy and 15 Gy, respectively [26].

Of note, the patient with fatal hemoptysis in our cohort presented with an elevated mean dose to the PBV (mean dose to 1 and 4 cc were of 53 and 50 Gy, respectively). Other dose constraints used by previous authors during thoracic reirradiation were reviewed by De Bari et al and corresponded with the normal tissue constraint guidelines from RTOG 0813 [12].

Another solution to decrease toxicities could be to increase the number of fractions during SABR, such as 8 fractions instead of 3 or 5, as already performed by Temming et al [27]. In the context of thoracic reirradiation, Liu et al encourages caution to be taken if patients are PS 2–3, have a forced expiratory volume in 1 s (FEV1) < 65%, previous PTV spanning bilateral mediastinum or V20 ≥ 30% on the composite plan (SABR + CFRT) [14].

One of the limitations of this present study is its retrospective nature and there was no prospective follow-up of the toxicities. We collected the toxicities reported in the records which can underreport low grades toxicities. To minimize this, a phone call was made to all living patients (*n* = 27) to collect missing toxicities. We could not reach 7 patients, which may lead to a recall bias. Due to the retrospective nature of the study (longstanding irradiation or previous irradiation performed in another center) we also could not perform cumulative dosimetry to better appreciate the risk of toxicity. Another limitation of this study is the absence of margin between GTV and CTV: this policy was used in order to decrease the risk of toxicities but this could have led to decreased tumor coverage.

Lastly, although our series includes the largest population of stage III patients reirradiated with SABR, the statistical power may not have been sufficient to detect all putative prognostic factors.

## Conclusions

This study reports long term efficacy and toxicity after SABR reirradiation in 46 patients with locally relapsed stage III NSCLC. Clinical outcome was interesting with a median PFS of 9.6 months and a median OS of 21.8 months. Care must be taken for an in-field relapse and central tumors due to their higher risk of toxicity.

## Additional file

Additional file 1: **Table S1.** Characteristics of patients with grade 5 toxicities. (DOCX 15 kb)

## Abbreviations

3D-CRT: Three dimensional conformal radiation therapy
BED: Biologically effective dose
CFRT: Conventionally fractionated radiation therapy
CR: Complete response
GTV: Gross tumor volume
LC: Local control
LRC: Locoregional control
MFS: Metastasis free survival
NSCLC: Non-small cell lung cancer
OS: Overall survival
PBV: Proximal bronchial vascular tree
PD: Progressive disease
PFS: Progression-free survival
PR: Partial response
PTV: Planning tumor volume
RECIST: Response evaluation criteria in solid tumors
SABR: Stereotactic ablative radiation therapy
SD: Stabilized disease
SHR: Subhazard ratio
