# Supplementary material for: Long term efficacy and toxicity after stereotactic ablative reirradiation in locally relapsed stage III non-small cell lung cancer
Source: BMC Cancer. 2019 Apr 3;19:305. doi: 10.1186/s12885-019-5542-3 (PMC6448259; doi:10.1186/s12885-019-5542-3)
Supplement: Supplementary file 1 — Table S1. Characteristics of patients with grade 5 toxicities. (DOCX 15 kb) [file 12885_2019_5542_MOESM1_ESM.docx]

| **Characteristics of patients with grade 5 toxicities** | | |
| --- | --- | --- |
|  | **Patient 1** | **Patient 2** |
| **Primary** | Right superior lobe | Left hilar – Left superior lobe |
| **3DCRT** | 20 x 2.25 Gy | 33 x 2 Gy |
| **Relapse** | Right Paratracheal | Left superior lobe |
| **SABR** | 5 x 10 Gy | 5 x 9 Gy |
| **Cause of death** | Pneumonitis | Hemoptysis |
|  | | |
| **Variable  (dose constraint)** |  |  |
| **Aorta** Dmax (< 53 Gy) 10 cc (< 47 Gy)  **Right pulmonary artery** Dmax (< 53 Gy) 10 cc (< 47 Gy)  **Left pulmonary artery** Dmax (< 53 Gy) 10 cc (< 47 Gy)  **Superior vena cava** Dmax (< 53 Gy) 10 cc (< 47 Gy)  **Oesophagus** Dmax (< 35 Gy) 5 cc (< 19.5 Gy)  **Spinal Cord** Dmax (< 30 Gy) 0.35 cc (< 23 Gy) 1.2 cc (< 14.5 Gy)  **Trachea** Dmax (< 40 Gy) 4 cc (< 16.5 Gy)  **Right proximal bronchus** Dmax (< 40 Gy) 4 cc (< 16.5 Gy)  **Left proximal bronchus** Dmax (< 40 Gy) 4 cc (< 16.5 Gy)  **Lungs** 1500 cc  (mean < 12.5 Gy) 1000 cc  (mean < 13.5 Gy)  **Right proximal bronchial vascular tree** 1 cc (< 20 Gy) 4 cc (< 15 Gy)   **Left proximal bronchial vascular tree** 1 cc (< 20 Gy) 4 cc (< 15 Gy) | 24.7 Gy 7.1 Gy    5 Gy 2.1 Gy    - -   61.6 Gy 32.2 Gy   9.2 Gy 3.9 Gy   4.8 Gy 3.5 Gy 2.8 Gy   8.8 Gy 5.3 Gy    5.5 Gy 3.2 Gy    - -   2 Gy  2 Gy      4.5 Gy 3 Gy     - - | 18.6 Gy 9.1 Gy    - -    45.5 Gy 25.6 Gy   7.2 Gy 3.5 Gy   9.2 Gy 5.3 Gy   3.7 Gy 3.4 Gy 3.2 Gy   9.8 Gy 6.1 Gy    5.1 Gy 2 Gy    31.2 Gy 8.8 Gy   2.1 Gy  3 Gy      - -     53 Gy 50 Gy |
| - : Out of field | | |
